# Comparing the Normalized Difference Vegetation Index with the Google Street View Measure of Vegetation to Assess Associations between Greenness, Walkability, Recreational Physical Activity, and Health in Ottawa, Canada

**DOI:** 10.3390/ijerph15081719

**Published:** 2018-08-10

**Authors:** Paul J. Villeneuve, Renate L. Ysseldyk, Ariel Root, Sarah Ambrose, Jason DiMuzio, Neerija Kumar, Monica Shehata, Min Xi, Evan Seed, Xiaojiang Li, Mahdi Shooshtari, Daniel Rainham

**Affiliations:** 1 Department of Health Sciences, Carleton University, Ottawa, ON K1S 5B6, Canada; renate.ysseldyk@carleton.ca (R.L.Y.); ArielRoot@cmail.carleton.ca (A.R.); VyjayanthiAmbrose@cmail.carleton.ca (S.A.); JasonDimuzio@cmail.carleton.ca (J.D.); NeerijaKumar@cmail.carleton.ca (N.K.); MonicaShehata@cmail.carleton.ca (M.S.); min.xi@mail.utoronto.ca (M.X.); 2 Dalla Lana School of Public Health, University of Toronto, Toronto, ON M5T 3M7, Canada; Evan.Seed@canue.ca; 3 Department of Urban Studies and Planning, Massachusetts Institute of Technology, Cambridge, MA 02139, USA; xiaojian@mit.edu; 4 Department of Geography, University of Victoria, Victoria, BC V8W 2Y2, Canada; mahdi.shooshtari@canue.ca; 5 Healthy Populations Institute, Dalhousie University, Halifax, NS B3H 4R2, Canada; Daniel.Rainham@Dal.Ca

**Keywords:** built environment, walkability, greenness, recreational physical activity, mental health, physical health

## Abstract

The manner in which features of the built environment, such as walkability and greenness, impact participation in recreational activities and health are complex. We analyzed survey data provided by 282 Ottawa adults in 2016. The survey collected information on participation in recreational physical activities by season, and whether these activities were performed within participants’ neighbourhoods. The SF-12 instrument was used to characterize their overall mental and physical health. Measures of active living environment, and the satellite derived Normalized Difference Vegetation Index (NDVI) and Google Street View (GSV) greenness indices were assigned to participants’ residential addresses. Logistic regression and least squares regression were used to characterize associations between these measures and recreational physical activity, and self-reported health. The NDVI was not associated with participation in recreational activities in either the winter or summer, or physical or mental health. In contrast, the GSV was positively associated with participation in recreational activities during the summer. Specifically, those in the highest quartile spent, on average, 5.4 more hours weekly on recreational physical activities relative to those in the lowest quartile (*p* = 0.01). Active living environments were associated with increased utilitarian walking, and reduced reliance on use of motor vehicles. Our findings provide support for the hypothesis that neighbourhood greenness may play an important role in promoting participation in recreational physical activity during the summer.

## 1. Introduction

More than half of the world’s population lives in urban areas, and this proportion continues to increase [1]. Urbanization is prevalent in Canada, where 82% of Canadians live in cities [2]. Between 1971 and 2001, in Canadian census metropolitan areas, the amount of space characterized by impervious surfaces (e.g., roadways, parking lots and roof tops) has increased from 5651 to 14,546 km^2^ [3]. Over the past decade, there has been an increasing awareness of the population health impacts of the features of built environment in urban areas.

Pedestrian-friendly, or walkable, neighbourhoods that facilitate access to local amenities and well-designed public open space has potential health benefits [4,5]. These benefits may be realized through increased levels of both utilitarian and recreational physical activity, which in turn reduce risks of obesity and chronic disease. A recent meta-analysis of approximately one hundred publications concluded that safe, walkable, and aesthetically pleasing places positively influenced participation in physical activities among older adults [6]. However, the review noted inconsistencies between the strength of the association depending on what methods were used to measure physical activity and the features of the built environment.

Previous work suggests that neighbourhood walkability confers benefits on residents’ physical health [7,8]. Cross-sectional analyses of the Multi-Ethnic Study of Atherosclerosis data found that walkability was associated with overall self-reported physical health, but not mental health [9]. In contrast, a more recent study in Hong Kong found that walkability was a statistically significant positive predictor of both mental and physical health [10]. Few studies have investigated neighbourhood walkability and subjective measures of well-being. These associations were examined in recent analyses of the US Behavioral Risk Factors Surveillance Survey, which found that those who lived in more walkable neighbourhoods were more likely to report improved overall general health, but were less satisfied with life [11]. Importantly, cross-sectional and quasi-longitudinal analyses of movers and non-movers that were able to account for neighbourhood preferences and individual attitudes found that neighbourhood design characteristics are associated with physical activity [12].

Evaluating the impacts of the built environment on physical activities in many counties, including Canada, is complicated by seasonal variations in temperature and other climatic conditions. The colder weather and snow that frequently occur during winter may be a deterrent to performing many recreational activities. For example, a survey of adults in Calgary, Canada found increased participation in moderate physical activity in the spring, summer, and autumn months compared to winter [13]. Apart from seasonal impacts on the overall participation in recreational physical activity, less ideal meteorological conditions could motivate individuals to participate in recreational activities closer to home due to increased challenges with mobility. For example, recent research in Vancouver, Canada found that neighbourhoods that have longer block lengths, fewer intersections, and are a greater distance from amenities are more likely to become inaccessible due to snow [14]. This same study also reported that older adults who live in walkable neighbourhoods walked to 25% fewer destinations when there was snow [14]. While some Canadian studies have adjusted for meteorological conditions when assessing how features of the built environment impact physical activity [15], to our knowledge no study has performed season-specific analyses. 

Similar to walkability, previous research that has studied neighbourhood availability to greenspace and physical activity suggests that greenspace is positively associated with physical activity [16]; however, positive associations have not been observed in all studies [17,18]. A national Canadian study reported that residential greenness was associated with increased participation in physical activity, and this pattern was evident across different income levels [19]. Most of these studies relied on a residentially-based measure of greenness, however, were unable to determine participants’ activity levels within their own neighbourhoods. We contend that it is important to capture where residents are physically active, as the benefits for those who live in greener and more walkable neighbourhood are more likely to be close to home. Findings from a recent study in Montreal, Canada support this hypothesis. Specifically, they found that neighbourhood walkability was associated with physical activities done within participants’ immediate neighbourhood but not with higher overall physical activity [20]. In addition to the possible impacts that access to greenspace may have on physical activity, there is a growing literature that suggests that greenspace may confer mental health benefits [21,22,23], and reduction in stress [24,25]. 

Previous epidemiological studies of greenness and health have predominantly relied on characterizing greenness using the Normalized Difference Vegetation Index [26]. The NDVI relies on satellite-derived images of the landscape that can measure greenness from above, including grasses, bushes, and tree canopies. This metric has limited use in epidemiological studies as it is unable to distinguish between different types of vegetation which may be relevant for some of the proposed underlying pathways related to health benefits of greenness [27]. The use of street view, rather than overhead measures of greenness may represent an improved metric as it better captures individuals’ perception of greenness. The street view measure can better capture dimensions of trees and other types of vegetation from a ground-based view. While there have been efforts to develop greenness metrics from Google Street View (GSV) images, to date they have not been applied to epidemiological data [28].

To address these research gaps, we undertook a cross-sectional study in Ottawa, Canada. Our specific aims were to evaluate associations between neighbourhood walkability and greenness and recreational physical activity and overall mental and physical health. These objectives include efforts to evaluate the extent to which associations vary by season, and neighbourhood-based participation in recreational physical activity. 

## 2. Materials and Methods

### 2.1. Study Population and Questionnaire

The data presented herein were collected as part of the BEYOND (Built Environments: Your Ottawa Neighbourhood and Determinants of Health) Study. The target population was adult residents within the city of Ottawa; however, the project was particularly interested in how features of the built environment impacted seniors, parents with young children, and those with mobility restrictions. As a result, these groups were overrepresented in our recruitment efforts, and the study population is not completely representative of Ottawa adult residents. 

We developed an online survey that was completed by participants over a two month time frame between January and March 2016. The survey was advertised through common public meeting spaces in different neighbourhoods in the Ottawa region, as well as online. The survey took, on average, 20 min to complete, and participants were offered to be placed in a random draw for gift cards as an incentive for them to complete the survey. In total, 447 survey respondents started the online survey; however, only 282 completed the survey having the age and place of residency inclusion criteria. We applied the place of residency inclusion criteria by restricting to those participants who provided a six character postal code that was located within the Ottawa census metropolitan area. In Canadian urban settings, a six character postal code typically represents one side of a street of a city block, or a single apartment building. Therefore, in Canadian urban areas six character postal codes have high locational accuracy. The online survey instrument we developed consisted of four sections: general socio-demographic characteristics, individuals’ perceptions of their neighbourhoods, self-reported health, and participation in recreational physical activities.

The socio-demographic section of the survey collected information on participants’ age, sex, household income, education, marital status, and employment status. Individuals were also asked to provide information related to their mobility restrictions, including the use of assistive devices (e.g., walking canes, and wheelchairs). The second section asked participants to describe features of their neighbourhoods including the types of spaces (e.g., developed, parks), public amenities (e.g., benches, bike stations, etc.), nearby recreational facilities, quality of sidewalks, characteristics of traffic intersections, and their use of public transportation. The third section used the SF-12 survey instrument to characterize participants’ overall physical and mental health. The SF-12 [29] is an abridged version of the 36-item Short Form Health Survey (SF-36) that was developed to measure health-related quality of life [30]. It contains eight different subscales of physical and mental functioning: role limitations due to physical problems, bodily pain, general health perceptions, vitality, social functioning, role limitations due to emotional problems, and mental health. We opted to use the second version of the SF-12 in our study as it has more focus on two distinct overall physical and mental health concepts, which are referred to as the Physical Component Summary (PCS) and the Mental Component Summary (MCS) [31]. We derived summary scores for the PCS and MCS for each survey participant using a previously described approach [32], and our calculations incorporated previously published Canadian normative data [33]. In the fourth survey section, for physical activity, individuals were asked to indicate how many hours they spent doing leisure-time physical activities both overall, and within their neighbourhood. Participants were told to consider their neighbourhood as the distance they could travel with a 20 min walk in any direction from their home. The specific question they were asked was: “How many hours per week did you spend doing leisure physical activities (i.e., walking, biking, skating, gardening)?”. They were asked to provide estimates for both the summer and winter. Finally, participants were asked to indicate how often they used car, walking, cycling, and public transit as a mode of transportation within the city. Possible responses to this question were: never, 1–3 times per month, once per week, 2–6 times per week, and more than 6 times per week. Finally, participants were asked to indicate their level of satisfaction with the overall quality of their neighbourhood and safety. Possible responses were very dissatisfied, dissatisfied, neutral, satisfied and very satisfied. 

### 2.2. Assignment of Walkability of Canadian Communities and Greenness

#### 2.2.1. Walkability

In Canada, the Walk Score^®^ is a commonly used measure to describe the walkability of a neighbourhood (www.walkscore.com). The Walk Score^®^ is an amenity-based measure of walkability where the score is determined based on the distance to amenities in each category, with higher scores awarded to those amenities within a 5 min walk (0.25 miles/0.4 km). In contrast, this study makes use of a GIS-based metric that focusses on the characteristics of the urban infrastructure. Specifically, we used a recently developed Canadian dataset that captures the active living environment (e.g., walkability of Canadian communities) that supports physical activity. The Canadian Active Living Environments (Can-ALE) database was designed for research on the design of communities and physical activity levels of their residents [34]. The Can-ALE measures were developed for the 2016 Canadian census dissemination areas. We modelled an index of active living environments that incorporated the following four measures: intersection density, dwelling density, local points of interest, as well as transit measures. The intersection density measure describes how direct and connected the streets and paths are within a community. It is calculated by counting the number of three (or more) way intersections within a 1 km buffer of the centroid of the dissemination area. The dwelling density measure captures how many dwellings are within a 1 km buffer of the centroid of the dissemination area. The points of interest measure is based on the number of points of interest with a 1 km buffer from the centroid of the dissemination area. Points of interest include examples such as: parks, schools, shops, places of business and landmarks, and this measure is strongly associated with active transportation rates. Finally, the transit measure captures the number of public transit stops in the community. The *Z*-scores across these four measures were used to derive a summary continuous measure of active living environments, and in turn, assigned at a census dissemination level. A *Z*-score of 0 would imply that the dissemination area is near the Canadian average for this measure. In Canada, a dissemination area is the smallest geographic area for which census data can be disseminated. Dissemination areas cover all of Canada, and these areas capture a population between 400–700 individuals [35]. The measures at a dissemination area were then linked to the 6 character postal codes of the participants using the CanMap Postal Code Suite program (DMTI Spatial, Richmond Hill, Ontario, Canada) [36]. The Can-ALE and Walk Score^®^ were moderately correlated for participants’ residential location (Pearson Correlation Coefficient = 0.78).

#### 2.2.2. Satellite Measure of Greenness—The NDVI

Our study used both a satellite and a street view measure of greenness. For the satellite-derived measure, the Normalized Difference Vegetation Index (NDVI), which is the most commonly used measure of ground-level vegetation [26], was calculated for each participant’s residence. Our NDVI measure was constructed using data originally generated by MODIS satellite sensors, which were then used to create a continuous measure of greenness for the Ottawa-area. MODIS data were acquired from the National Aeronautics and Space Administration’s Earth Observing System Data and Information System (EOSDIS) using the web-based tool called Reverb. The image chosen originated from the MODIS Aqua satellite, which carries the same sensor as the MODIS Terra. The NDVI image was derived from MODIS optical data by operators at NASA (from imagery corrected for atmospheric interference) using near-infrared, red and blue bands. The dataset contained 16-day average vegetation indices at a 250 m × 250 m resolution for the 2011 summer (August) season. A spatial filter was applied to the data to generate a cloud-free, quality NDVI surface with minimal residual atmospheric contamination. The ‘mosaic’ function in ArcMap GIS software (Environmental Systems Research Institute: Redlands, CA, USA) [37] was used to create a continuous surface and the data were re-projected to Albers Equal Area Conic projection so that distortion of areas containing NDVI cell values could be minimized. The Albers Equal Area Conic uses two standard parallels to reduce distortion and assigns coordinates as eastings and northings in meters. It has been estimated that the maximum scale distortion for large continental land masses in North America is 1.3 percent. The residential measure of greenness we used corresponds to the NDVI value for the raster cell in which centroid of the six character postal code resides. Since the resolution of the NDVI data is 250 m then the value would represent the average NDVI value for a 250 m × 250 m area around the geocoded postal code location. Values closer to “1” are representative of areas with more vegetation, while values near “0” are representative of areas with impervious surfaces.

#### 2.2.3. Street View Measure of Greenness—The GVI

We also assigned the Green View Index (GVI) to participants’ place of residence. The GVI measures the amount of vegetation along city streets and is computed using Google Street View (GSV) images [38]. The GVI ranges in value from 0 to 100 and provides an indicator of the percentage of vegetation as viewed from a street location. We submitted a set of geographical coordinates corresponding to the postal codes of participants’ residences to the GSV Image API to collect images. GSV images were captured in 6 different directions so as to cover the full panorama view for each residence using methodology previously described [28]. Of the 282 sample locations with a postal code, 17 did not return GSV images or returned indoor GSV images that were not valid for the calculation of the GVI. The estimation of greenness classification was then performed using segmentation techniques that identify green objects within the images. The GVI was computed for all 6 images at each location, and these values were then averaged to produce a single GVI value for location. In Ottawa, images captured outside the growing season were excluded. Specifically, we only included images during the months between June and September which correspond to the time of the year when trees still have their leaves. 

### 2.3. Statistical Analyses

The Statistical Analysis System version 9.4 (SAS Institute, Cary, NC, USA) was used to perform all data analyses. A dot plot was generated to depict the geographic locations of participants’ residences within Ottawa. For this figure alone, place of residence locations were randomly moved to a distance of 100–200 m to protect the confidentiality of the study participants. Descriptive analyses were done to describe the survey sample by socio-demographic factors, and present the mean greenness and walkability measures across these categories. Analysis of Variance methods were used to test for statistically significant differences in these means across categories. Boxplots were created to describe the distribution of the self-reported physical (PCS) and mental health (MCS) component scores, and weekly hours spent on recreational activities in the summer and winter seasons.

Least squares adjusted means were used to describe associations between the measures of physical activity, and the PCS and MCS scores for neighbourhood measures of (i) active living environment; (ii) satellite measures of greenness (NDVI); and (iii) the street view measure of greenness (GVI). For all models, features of the built environment were examined as the independent variable, and the health measures represented the dependent variables whose means were compared across categories. For walkability and greenness, we categorized neighbourhood measures into quartiles based on the frequency distribution of these variables. Spline analyses were undertaken to characterize the relationship between the street view measure of greenness (GVI) and recreational physical activity levels in the summer. Finally, to examine the associations between the neighbourhood measures of the built environment and utilitarian forms of transportation, we conducted least squares regression analyses against participants’ self-reported frequency of different forms of transportation. These included: transport by car, walking, cycling, and public transportation.

## 3. Results

**Figure 1:**
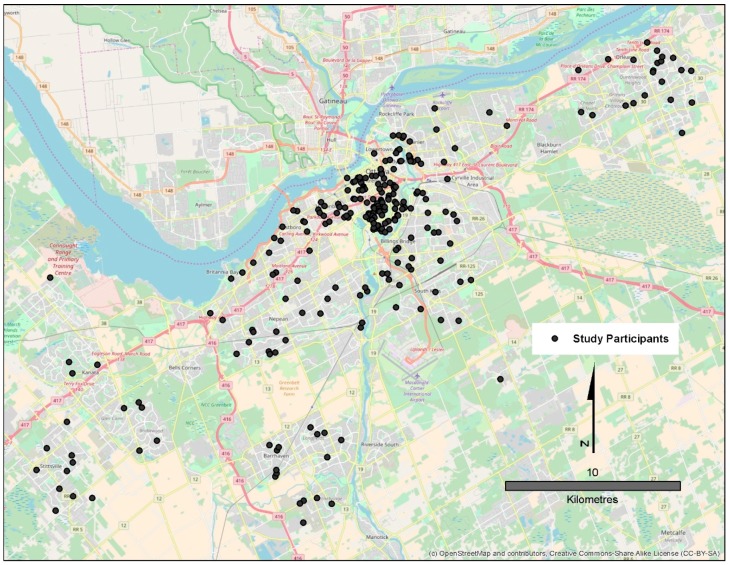
Approximate residential locations of participants of the BEYOND study, Ottawa, Canada.

In total, 447 adults responded to the invitation to participate in the survey, and of these 331 completed the online survey, and consented for allowing their data to be used. After excluding those who lived outside the Ottawa region as well as those who did not provide a residential postal code, data from 282 participants were retained for analyses. The approximate (<200 m) geographical locations of these participants are displayed in Figure 1. Nearly one-quarter (28.0%) of participants resided in the postal codes in the region located just south of the downtown core; relatively few respondents resided in the suburbs of Ottawa.

Sixty-two percent of the participants were female. The mean age of participants was 41.6 years, and the age of participants ranged from 18 to 77 years of age. We found no association between the activity living environment (ALE) score and the age, sex, or marital status of the participants (Table 1). However, this index was lowest among those with household incomes over $125,000 (*μ* = 1.91), as well as among parents of children less than 5 years of age (*μ* = 1.95). In contrast to walkability, both age and income were positively correlated with the overhead measure of neighbourhood greenness. Among participants 56 years of age and older, the mean NDVI score was 0.58 (95% CI = 0.52–0.64), while the corresponding estimate among those between the ages of 18 and 28 was 0.53 (95% CI = 0.47–0.62). Similar patterns were observed with household income and NDVI.

**Figure 2:**
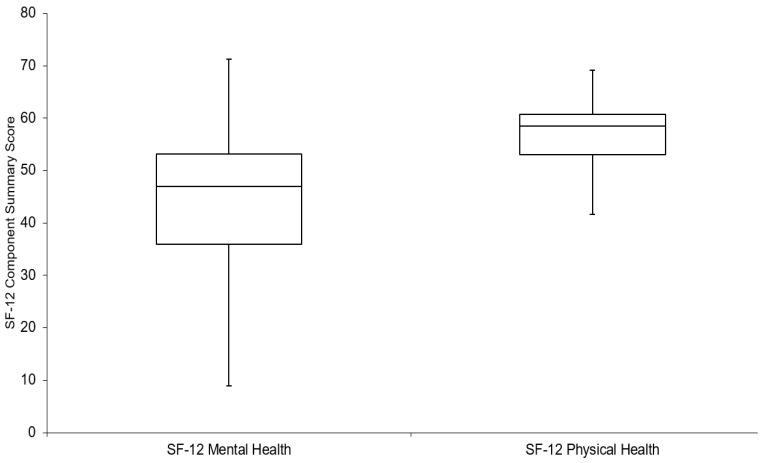
Boxplots depicting distribution of SF-12 Mental and Physical Health Component Summary Scores.

The frequency distributions of our outcomes of interest are presented in boxplots (Figure 2). For the mental health component summary score of the SF-12, the overall mean score was 48.8 (s.d. = 11.8), while for the physical health component measure it was 55.0 (s.d. = 10.0). Participants reported spending more time on recreational physical activities during the summer when compared to winter. Specifically, during the summer season, participants reported spending 11.9 h weekly on recreational physical activity compared to 7.7 h during the winter season (*p* < 0.05). In the winter, participants reported that, on average, 60% of the weekly number of hours of recreational activities were spent within their neighbourhood. The corresponding estimate was slightly higher in the summer (70%).

The mean number of hours spent on recreational physical activities across neighbourhoods across quartiles of the active living environment index are presented Table 2. Overall, we found no statistically significant differences in the number of hours spent on recreational activities during either the summer or winter by neighbourhood walkability. However, when we restricted analyses to recreational physical activities performed within participants’ immediate neighbourhoods during the winter season, those who lived in neighborhoods in the upper quartile of active living environments, on average, spent 1.4 h more on recreational activities in their neighbourhoods when compared to those in the lowest quartile (*p* = 0.03). We observed no clear associations between the active living environment index with either physical or mental health measures. 

**Figure 3:**
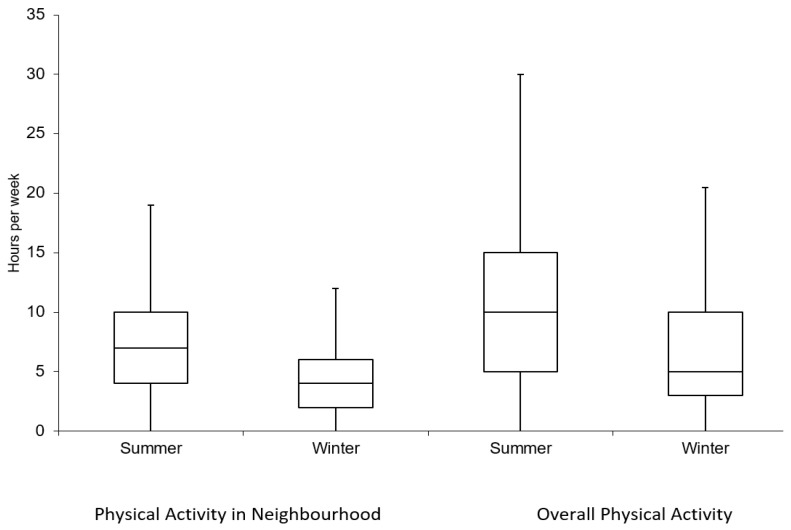
Boxplots depicting participation in Recreational Physical Activities within participants’ neighbourhoods and overall, by season.

**Figure 4:**
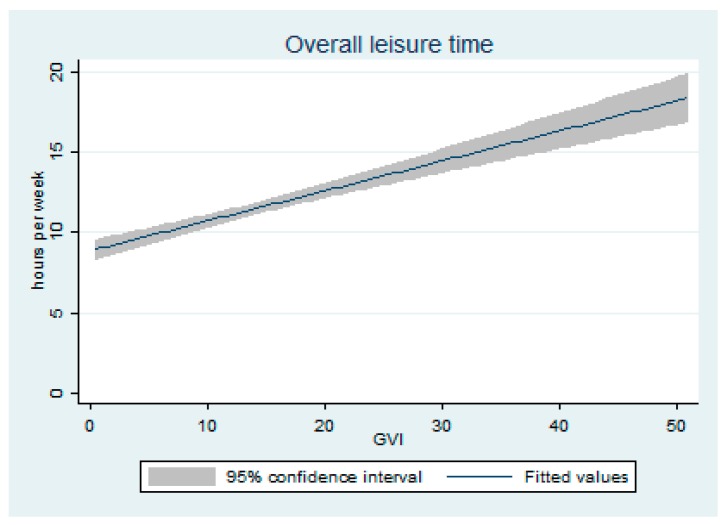

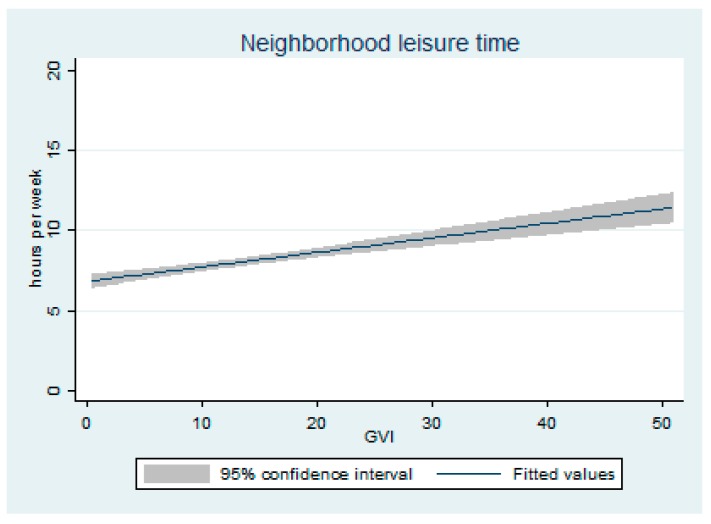
Associations between street view measures of greenness (Green View Index) and weekly hours of recreational physical activity during the summer, BEYOND participants. The curves were generated from spline analyses and were adjusted for age, sex income, marital status, having a young child in household, number of years of residency in neighbourhood, and perceived neighourhood safety.

We found no statistically significant associations between the satellite-derived measure of greenness, the NDVI, and physical activity, Physical Component Summary (PCS), or Mental Component Summary (MCS) scores (Table 3). In contrast, we found statistically significant associations between street view measures of greenness, the GVI, and the number of hours of recreational activity during the summer (Table 4; *p* = 0.01). Overall levels of recreational activity were higher in the summer when compared to the winter (Figure 3). Those who lived in the highest quartile of greenness based on the GVI spent on average 18.1 h on recreational activities each week, while those in the lowest quartile spent 12.7 h. A similar pattern was observed when we examined recreational activities performed within participants’ neighbourhoods, but this finding was not statistically significant (*p* = 0.13). Spline analyses of these associations revealed a linear trend between the GVI and number of weekly hours of recreational activity during the summer (Figure 4). The GVI was not associated with the number of hours spent weekly on recreational activities during the winter, nor with the PCS or MCS.

Transportation use was related to the neighbourhood Active Living Environment (ALE) (Table 5). In particular, participants who were likely to use their cars daily had lower Can-ALE score when compared to those who used their car less frequently (*p* < 0.001). In contrast, the mean Can-ALE score for those who did not use walking as a form of transportation was 1.33, while for those who walked daily the mean score was 4.45. No statistically significant associations in these scores were observed for different frequencies of utilitarian cycling or use of public transportation. Neither metric of greenness was a statistically significant predictor of transportation use.

## 4. Discussion

Using a cross-sectional study, we examined the roles of neighbourhood greenness and walkability on participation in recreational physical activities, and self-reported mental and physical health in Ottawa, Canada. As Ottawa has substantial variations in climate over the course of the year, our survey was designed to capture associations with physical activity in both the winter and the summer. In Ottawa, the daily mean temperature during the summer time is approximately 20 °C, while dropping down to approximately −10 °C during the winter months of January and February. We found that there was no association between satellite-derived neighbourhood measures of greenness, with either participation in physical activity or for self-reported measures of health. However, objectively-defined street view measures of greenness were positively associated with participation in recreational activities during the summer, but not winter season. This study represents one of the first attempts to look at seasonal variations between features of the built environment and physical activity in a Canadian city. 

Similar to a number of other studies [17,18], we found no association between overhead neighbourhood measures of greenness and participation in physical activity. Our analyses relied on the use of the NDVI exposure metric. While this has been the most commonly used metric in previous epidemiological studies of greenness, it has several limitations. As others have noted, it is unable to distinguish between different types of natural space, nor is it capable of describing the accessibility of areas [16,39]. The relatively small size of our study sample did not allow us to perform stratified analyses across these other features of green space. However, our findings were essentially unchanged after adjusting for socio-demographic characteristics, or perceived safety of the neighbourhood. Further efforts to enhance the performance of the NDVI to account for additional greenspace characteristics are needed to provide additional insights on which features are most important for enhancing participation in physical activity. While the GVI metric we applied to the data better capture individuals’ perceptions of vegetation from a street view, more work is needed to enhance this metric to better describe other green space features such as quality, and accessibility. An important limitation of the GVI is that it only captures greenness from the street view, and is unable to characterize greenness in participants’ properties, particularly their backyards. Other related work to enhance measures of greenness that is ongoing includes efforts by investigators in Vancouver, Canada to develop the Natural Space Index [40]. 

The collection of qualitative data from study participants can yield important insights on characteristics of the built environment that may be barriers to participation in physical activity. The BEYOND study did conduct focus groups of a small number of participants (*n* = 14) but the aim of these groups was to identify what changes to the neighbourhood were desired by those with mobility restrictions, seniors, and parents with young children. Nonetheless, these participants did identify that sidewalk design and maintenance were key concerns. Parents with young children indicated that well maintained streets facilitated year round active transportation. In contrast, those with mobility restrictions indicated that poor street maintenance impacted safety as well as accessibility. Seniors, in contrast, highlighted the vital role that proximity to resources has on maintaining social connectedness to others. The qualitative data highlight that there are key differences in what aspects of the built environment are important within urban populations.

A strength of our study was our ability to capture participation in recreational physical activities within participants’ neighbourhoods. This overcomes past limitations of previous national analyses that have been published [19]. During the summer season, we found a stronger association between residential street view measures of greenness and overall recreation when compared to the association observed with recreational activities done within participants’ neighbourhood. These comparisons were somewhat limited due to small sample size, and could reflect differences in the types of activities performed. Future Ottawa based work should be extended to capture participation in specific recreational activities at venues outside individuals’ neighbourhoods. Recent investigations have incorporated objectively collected data using global positioning systems and accelerometry data to better understand whether individuals are physically active in greener areas [41,42,43]. In Chino California, children who spent 20 min per day in greener areas expended 5 times the daily rate of moderate to vigorous physical activities relative to those who spent no time in green areas [42]. In contrast, a study of 180 middle-aged adults in Ghent, Belgium found associations between spending more time in green areas and increased levels of physical activity; however, these associations varied by sex, and educational attainment [41]. Finally, analyses of GPS and accelerometry data from adult women across the US found that higher levels of physical activity occurred in greener and more walkable areas, and associations were most pronounced among those who were white and more affluent [43]. While the use of personal monitoring devices to capture activity patterns by location can provide important insights on how features of the built environment influence physical activity, the application of these exposure strategies to studies with a large number of participants remain impractical at this time. Nonetheless, they should be pursued particularly at a local level, and supplemented with qualitative research, including focus groups, to better understand barriers to participating in recreational physical activities that vary within and between cities. Recent work in Calgary suggests that there is an extensive number of local factors that influence participation in physical activity including: quality and length of sidewalks, number of paths, population density, and tree density [44].

Overall, there was little association between the measure of active living environment and physical activity, apart from a hint of increased participation in recreational activities during the winter season. The same pattern was also observed for the amenity-based Walk Score^®^ metric. It is important to note that our physical activity outcome was restricted to recreational physical activity. However, our findings related to our measures of the built environment and frequency of using active transportation suggest that those living in neighbourhoods with higher active living environments are more likely to walk and cycle, while less likely to rely on cars for transportation. In contrast, measures of greenness had little impact on utilitarian cycling and walking. 

This study has a number of limitations. Like other studies of greenness and physical activities, positive associations may be a reflection of self-selection bias. Namely, those of greater affluence, or those who are more physically active, may choose to live in neighbourhoods with greater access to parks, or larger tree canopies. Longitudinal studies are needed to better understand whether changes in the built environments, including greenness and active living environments, can help reduce sedentary behaviours. The BEYOND Study used an online survey, and while we attempted to recruit participants from across the city of Ottawa by advertising in centers across the city, caution should be exerted when trying to generalize these findings to the general population. The online nature of the survey precluded an estimation of participation rates. We conducted the study in consultation with local public health officials who had a particular interest in understanding how urban design might impact those with mobility limitations, the elderly, and young families. Our study population reflects this. However, the participants were not made aware of our specific intent to look at measures of greenness and active living on physical activity, and therefore, in our view, our findings are unlikely to be biased due to self-reported responses. 

## 5. Conclusions

In summary, our analyses suggest that, in Ottawa, street view based measures of greenness are associated with participation in recreational physical activity during the summer season. Importantly, the poor correlation observed between the NDVI and GVI (*r* = 0.24) suggests that these metrics capture different aspects of urban greenness. Active living environments were associated with increased utilitarian walking and reduced reliance on private automobile-based transportation. We are currently extending our research activities to include the evaluation of alternative measures of greenness, and natural spaces within a larger sample and more representative sample of Ottawa residents. 

It is now recognized that the health impacts of greenness are varied as several different health outcomes and pathways are involved. These pathways can include, for example, increased participation in recreational activities, increased social interactions, shelter from ultraviolet radiation, reductions in air pollution, noise and temperatures [16]. Our findings highlight the important need to consider different measures of greenness that best represent these pathways. 

## Figures and Tables

**Table 1:** Descriptive characteristics of BEYOND study participants, and neighbourhood measures of walkability and greenness.

Characteristic		Participants		NDVI			GVI			ALE		
		*n*	%	Mean	IQR	*p* *	Mean	IQR	*p* *	Mean	IQR	*p* *
Sex	Male	104	36.8	0.55	0.50–0.64	0.89	14.34	8.11–19.85	0.92	3.15	0.78–4.52	0.43
	Female	179	61.2	0.55	0.50–0.63		14.22	8.30–18.38		2.83	0.85–4.22	
Age-Group	18–28	72	25.4	0.53	0.47–0.62	0.01	14.49	9.50–17.46	0.32	3.26	0.70–5.49	0.58
	29–37	68	24.0	0.52	0.46–0.61		12.83	6.45–18.37		3.18	0.94–4.50	
	39–55	72	25.4	0.56	0.51–0.65		15.52	9.72–19.82		2.70	0.91–3.69	
	≥56	71	25.1	0.58	0.52–0.64		14.08	7.72–20.62		2.66	0.85–4.21	
Household Income (in CDN $)	<20,000	25	8.8	0.55	0.45–0.68	0.01	16.13	10.34–19.56	0.30	3.39	1.15–4.48	0.01
	20,000–<50,000	39	13.8	0.53	0.47–0.61		13.67	8.11–17.28		3.01	0.71–4.53	
	50,000–<80,000	57	20.1	0.52	0.48–0.58		13.27	7.70–16.63		3.19	0.89–4.99	
	80,000–<125,000	45	15.9	0.52	0.48–0.61		12.40	6.07–19.53		4.14	1.34–5.22	
	≥125,000	61	21.6	0.58	0.52–0.65		15.10	10.40–20.77		1.91	0.17–3.38	
	Unknown	56	19.8	0.59	0.53–0.65		15.52	9.36–18.54		2.62	0.91–4.12	
Attained Education	High school or less	29	10.3	0.54	0.51–0.63	0.09	13.72	10.41–17.46	0.42	2.05	0.70–2.70	0.25
	College/Undergrad	140	49.5	0.53	0.46–0.63		14.35	8.42–18.78		2.88	0.74–4.58	
	Graduate	98	34.6	0.58	0.52–0.63		14.80	7.88–20.81		3.21	1.03–4.51	
	Professional	15	15.0	0.56	0.54–0.60		10.84	3.04–16.63		3.83	2.50–4.48	
Marital Status	Married/Common law	172	61.0	0.56	0.51–0.63	0.07	14.18	7.72–19.68	0.47	2.77	0.89–4.18	0.27
	Separ/Divorced/Widow	23	8.2	0.56	0.51–0.61		12.59	8.97–15.57		2.54	0.55–4.53	
	Single	87	30.8	0.53	0.45–0.64		15.00	8.50–18.39		3.41	0.71–5.46	
Have Children < 5 years of age Under care	No	226	81.3	0.55	0.50–0.63	0.19	14.34	8.42–19.53	0.86	3.18	0.87–4.63	0.01
	Yes	52	18.7	0.57	0.51–0.63		14.57	8.39–18.78		1.95	0.04–3.77	
Years Lived in Neighbourhood	<5 year	92	32.5	0.54	0.50–0.60	0.34	13.25	7.18–17.56	0.30	3.80	1.09–5.60	0.01
	5–<10 year	94	33.2	0.55	0.48–0.64		14.28	7.72–18.87		2.30	0.27–3.73	
	≥10 year	97	34.2	0.56	0.52–0.63		15.21	9.64–20.62		2.77	0.94–4.20	
Total		283	100.0									

* The *p*-values were obtained from Analysis of Variance Models and tested for differences in the means across the categories. GVI = Green View Index; NDVI = Normalized Difference Vegetation Index; CDN = Canadian; ALE = Active Living Environment.

**Table 2:** Least squares adjusted means * for leisure time participation in physical activities, and SF-12 composite measures of physical and mental health, by neighbourhood active living environment index.

Health Measure	Units	Adjusted Means* for Neighbourhood Walkability Quartiles Based on Active Living Environment				*p*
		Lowest	Med-Low	Med-High	High	
Overall leisure time physical activity (summer)	Hours per week	13.6	13.9	12.3	14.0	0.77
Neighbourhood leisure time physical activity (summer)	Hours per week	8.1	8.2	7.7	8.0	0.96
Overall leisure time physical activity (winter)	Hours per week	6.2	6.6	6.6	6.0	0.78
Neighbourhood leisure-time physical activity (winter)	Hours per week	3.3	3.6	4.6	4.7	0.03
SF-12 Mental Component Summary	Norm mean = 50, SD = 10	39.8	35.7	39.0	38.9	0.38
SF-12 Physical Component Summary	Norm mean = 50, SD = 10	54.9	55.2	54.9	57.4	0.14

* Adjusted for age, sex income, marital status, having a young child in household, number of years of residency in neighbourhood, and perceived neighourhood safety.

**Table 3:** Least squares adjusted means* for leisure time participation in physical activities, and physical and mental health, based on a satellite-derived measures of greenness (NDVI).

Health Measure	Units	Adjusted Means * for Neighbourhood Greenness Quartile based on NDVI				*p*
		Lowest	Med-Low	Med-High	High	
Overall leisure time physical activity (summer)	Hours per week	12.1	11.5	13.8	12.1	0.28
Neighbourhood leisure time physical activity (summer)	Hours per week	6.5	7.1	8.5	7.0	0.14
Overall leisure time physical activity (winter)	Hours per week	6.9	5.8	7.1	4.8	0.47
Neighbourhood leisure-time physical activity (winter)	Hours per week	3.1	3.3	4.1	2.9	0.37
SF-12 Mental Component Summary	Normative values	37.1	33.0	37.7	36.2	0.37
SF-12 Physical Component Summary	Normative values	55.3	59.3	54.8	56.1	0.81

* Adjusted for age, sex income, marital status, having a young child in household, number of years of residency in neighbourhood, and perceived neighourhood safety.

**Table 4:** Least squares adjusted means* for leisure time participation in physical activities, and physical and mental health, by Green View Index.

Health Measure	Units	Adjusted Means * Neighbourhood Greenness Quartile based on the GVI				*p*
		Lowest	Med-Low	Med-High	High	
Overall leisure time physical activity (summer)	Hours per week	12.7	15.0	14.6	18.1	0.01
Neighbourhood leisure time physical activity (summer)	Hours per week	8.4	8.3	8.7	10.3	0.13
Overall leisure time physical activity (winter)	Hours per week	5.4	7.7	8.3	8.0	0.26
Neighbourhood leisure-time physical activity (winter)	Hours per week	3.4	4.1	4.5	3.7	0.67
SF-12 Mental Component Summary	Normative values	37.8	38.0	36.8	35.8	0.44
SF-12 Physical Component Summary	Normative values	55.9	55.0	56.5	55.7	0.36

* Adjusted for age, sex income, marital status, having a young child in household, number of years of residency in neighbourhood, and perceived neighourhood safety.

**Table 5:** Adjusted means * of the neighbourhood active living environment index (Can-ALE), satellite derived measures of greenness (NDVI), and Green View Index based on the frequency of different forms of transportation used.

Transportation Type	Frequency	Can-ALE		NDVI		GVI	
		Mean	*p*-Value	Mean	*p*-Value	Mean	*p*-Value
Car	Never	4.29	<0.001	0.57	0.11	8.00	0.42
	1–3 per month	6.20		0.56		9.07	
	Once per week	4.50		0.58		10.80	
	2–6 times per week	2.26		0.59		10.64	
	>6 times per week	1.96		0.63		10.96	
Walk	Never	1.33	0.0002	0.62	0.56	8.78	0.63
	1–3 per month	2.65		0.57		8.95	
	Once per week	0.95		0.59		12.21	
	2–6 times per week	3.67		0.61		10.27	
	>6 times per week	4.45		0.58		8.85	
Cycle	Never	2.66	0.43	0.58	0.17	10.49	0.34
	1–3 per month	3.06		0.57		8.56	
	Once per week	2.99		0.62		11.68	
	2–6 times per week	3.20		0.63		10.74	
	>6 times per week	4.38		0.61		7.35	
Public Transit	Never	2.43	0.15	0.60	0.54	9.71	0.95
	1–3 per month	3.76		0.60		9.56	
	Once per week	4.38		0.65		9.06	
	2–6 times per week	3.69		0.58		10.55	
	>6 times per week	2.75		0.57		8.59	

* Adjusted for age, sex, income, marital status, number of years lived in neighbourhood, having a young child in household, number of years of residency in neighbourhood, and perceived neighourhood safety.

## References

[1] Wigginton N.S., Fahrenkamp-Uppenbrink J., Wible B., Malakoff D. (2016). Cities are the Future. Science.

[2] Turcotte M. (2008). The city/suburb contrast: How can we measure it?. Can. Soc. Trends.

[3] Statistics Canada (2016). Human Activity and the Environment 2015: The Changing Landscape of Canadian Metropolitan Areas.

[4] Giles-Corti B., Timperio A., Bull F., Pikora T. (2005). Understanding physical activity environmental correlates: Increased specificity for ecological models. Exerc. Sport Sci. Rev..

[5] Van Cauwenberg J., De Bourdeaudhuij I., Clarys P., Nasar J., Salmon J., Goubert L., Deforche B. (2016). Street characteristics preferred for transportation walking among older adults: A choice-based conjoint analysis with manipulated photographs. Int. J. Behav. Nutr. Phys. Act..

[6] Barnett D.W., Barnett A., Nathan A., Van Cauwenberg J., Cerin E., Council on E., on behalf of the Council on Environment and Physical Activity (CEPA)—Older Adults working group (2017). Built environmental correlates of older adults’ total physical activity and walking: A systematic review and meta-analysis. Int. J. Behav. Nutr. Phys. Act..

[7] Haselwandter E.M., Corcoran M.P., Folta S.C., Hyatt R., Fenton M., Nelson M.E. (2015). The built environment, physical activity, and aging in the United States: A state of the science review. J. Aging Phys. Act..

[8] Norman G.J., Carlson J.A., O’Mara S., Sallis J.F., Patrick K., Frank L.D., Godbole S.V. (2013). Neighborhood preference, walkability and walking in overweight/obese men. Am. J. Health Behav..

[9] Tomey K., Diez Roux A.V., Clarke P., Seeman T. (2013). Associations between neighborhood characteristics and self-rated health: A cross-sectional investigation in the Multi-Ethnic Study of Atherosclerosis (MESA) cohort. Health Place.

[10] Zhao Y., Chung P.K. (2017). Neighborhood environment walkability and health-related quality of life among older adults in Hong Kong. Arch. Gerontol. Geriatr..

[11] Oishi S., Saeki M., Axt J. (2015). Are People Living in Walkable Areas Healthier and More Satisfied with Life?. Appl. Psychol. Health Well Being.

[12] Handy S.L., Cao X., Mokhtarian P.L. (2008). The causal influence of neighborhood design on physical activity within the neighborhood: Evidence from Northern California. Am. J. Health Promot..

[13] McCormack G.R., Friedenreich C., Shiell A., Giles-Corti B., Doyle-Baker P.K. (2010). Sex- and age-specific seasonal variations in physical activity among adults. J. Epidemiol. Community Health.

[14] Clarke P., Hirsch J.A., Melendez R., Winters M., Sims Gould J., Ashe M., Furst S., McKay H. (2017). Snow and Rain Modify Neighbourhood Walkability for Older Adults. Can. J. Aging.

[15] Katapally T.R., Rainham D., Muhajarine N. (2015). Factoring in weather variation to capture the influence of urban design and built environment on globally recommended levels of moderate to vigorous physical activity in children. BMJ Open.

[16] James P., Banay R.F., Hart J.E., Laden F. (2015). A Review of the Health Benefits of Greenness. Curr. Epidemiol. Rep..

[17] Hillsdon M., Panter J., Foster C., Jones A. (2006). The relationship between access and quality of urban green space with population physical activity. Public Health.

[18] Maas J., Verheij R.A., Spreeuwenberg P., Groenewegen P.P. (2008). Physical activity as a possible mechanism behind the relationship between green space and health: A multilevel analysis. BMC Public Health.

[19] McMorris O., Villeneuve P.J., Su J., Jerrett M. (2015). Urban greenness and physical activity in a national survey of Canadians. Environ. Res..

[20] Hajna S., Kestens Y., Daskalopoulou S.S., Joseph L., Thierry B., Sherman M., Trudeau L., Rabasa-Lhoret R., Meissner L., Bacon S.L. (2016). Neighbourhood walkability and home neighbourhood-based physical activity: An observational study of adults with type 2 diabetes. BMC Public Health.

[21] Dadvand P., Bartoll X., Basagana X., Dalmau-Bueno A., Martinez D., Ambros A., Cirach M., Triguero-Mas M., Gascon M., Borrell C. (2016). Green spaces and General Health: Roles of mental health status, social support, and physical activity. Environ. Int..

[22] Kim J., Kim H. (2017). Demographic and Environmental Factors Associated with Mental Health: A Cross-Sectional Study. Int. J. Environ. Res. Public Health.

[23] Bos E.H., van der Meulen L., Wichers M., Jeronimus B.F. (2016). A Primrose Path? Moderating Effects of Age and Gender in the Association between Green Space and Mental Health. Int. J. Environ. Res. Public Health.

[24] Pun V.C., Manjourides J., Suh H.H. (2018). Association of neighborhood greenness with self-perceived stress, depression and anxiety symptoms in older U.S adults. Environ. Health.

[25] Triguero-Mas M., Donaire-Gonzalez D., Seto E., Valentin A., Martinez D., Smith G., Hurst G., Carrasco-Turigas G., Masterson D., van den Berg M. (2017). Natural outdoor environments and mental health: Stress as a possible mechanism. Environ. Res..

[26] Fong K.C., Hart J.E., James P. (2018). A Review of Epidemiologic Studies on Greenness and Health: Updated Literature Through 2017. Curr. Environ. Health Rep..

[27] Nieuwenhuijsen M.J. (2015). Exposure Assessment in Environmental Epidemiology.

[28] Li X., Zhang C., Li W., Ricard R., Meng Q., Zhang W.U. (2015). Assessing street-level urban greenery using Google Street View and a modified green view index. Urban For. Urban Green..

[29] Ware J., Kosinski M., Keller S.D. (1996). A 12-Item Short-Form Health Survey: Construction of scales and preliminary tests of reliability and validity. Med. Care.

[30] Ware J.E., Sherbourne C.D. (1992). The MOS 36-item short-form health survey (SF-36). I. Conceptual framework and item selection. Med. Care.

[31] Ware J.E., Kosinski M., Turner-Bowker D.M., Gandek B. (2002). How to Score Version 2 of the SF-12 Health Survey.

[32] Fleishman J.A., Selim A.J., Kazis L.E. (2010). Deriving SF-12v2 physical and mental health summary scores: A comparison of different scoring algorithms. Qual. Life Res..

[33] Hopman W.M., Towheed T., Anastassiades T., Tenenhouse A., Poliquin S., Berger C., Joseph L., Brown J.P., Murray T.M., Adachi J.D. (2000). Canadian normative data for the SF-36 health survey. Canadian Multicentre Osteoporosis Study Research Group. CMAJ.

[34] Ross N., Wasfi R., Herrmann T., Gleckner W. (2018). Canadian Active Living Environments Database (Can-ALE) User Manual & Technical Document.

[35] Statistics Canada Dissemination Area: Plain Language Summary. https://www.statcan.gc.ca/pub/92-195-x/2011001/geo/da-ad/da-ad-eng.htm.

[36] DMTI Spatial Inc (2016). CanMap Postal Code Suite, v2016.3.

[37] Environmental Systems Research Institute (ESRI) (2016). ArcGIS Desktop: Release 10.4.

[38] MIT Senseable Lab Treepedia: Exploring the Green Canopy in Cities Around the World. http://senseable.mit.edu/treepedia.

[39] Fan Y., Das K.V., Chen Q. (2011). Neighborhood green, social support, physical activity, and stress: Assessing the cumulative impact. Health Place.

[40] Rugel E.J., Henderson S.B., Carpiano R.M., Brauer M. (2017). Beyond the Normalized Difference Vegetation Index (NDVI): Developing a Natural Space Index for population-level health research. Environ. Res..

[41] Dewulf B., Neutens T., Van Dyck D., De Bourdeaudhuij I., Broekx S., Beckx C., Van de Weghe N. (2016). Associations between time spent in green areas and physical activity among late middle-aged adults. Geospat. Health.

[42] Almanza E., Jerrett M., Dunton G., Seto E., Pentz M.A. (2012). A study of community design, greenness, and physical activity in children using satellite, GPS and accelerometer data. Health Place.

[43] James P., Hart J.E., Hipp J.A., Mitchell J.A., Kerr J., Hurvitz P.M., Glanz K., Laden F. (2017). GPS-Based Exposure to Greenness and Walkability and Accelerometry-Based Physical Activity. Cancer Epidemiol. Biomarkers Prev..

[44] McCormack G.R. (2017). Neighbourhood built environment characteristics associated with different types of physical activity in Canadian adults. Health Promot. Chronic Dis. Prev. Can..

